# AUF1 positively controls angiogenesis through mRNA stabilization-dependent up-regulation of HIF-1α and VEGF-A in human osteosarcoma

**DOI:** 10.18632/oncotarget.27115

**Published:** 2019-08-06

**Authors:** Huda H. Al-Khalaf, Abdelilah Aboussekhra

**Affiliations:** ^1^Department of Molecular Oncology, King Faisal Specialist Hospital and Research Center, Riyadh 11211, KSA; ^2^The National Center for Stem Cell Technology, King Abdulaziz City for Science and Technology, Riyadh 11211, KSA

**Keywords:** osteosarcoma, AUF1, VEGF-A, HIF-1α, angiogenesis

## Abstract

Osteosarcoma is the most common malignant bone tumor in children, adolescents, and young adults. This pleiomorphic tumor depends on new blood vessel development, also known as angiogenesis, for tumor growth and metastasis. Therefore, it’s of utmost importance to identify the key genes and pathways that regulate this pro-metastatic process in order to develop more efficient therapies. Here, we have shown that the RNA-binding protein AUF1 positively regulates the expression of the pro-angiogenic factor VEGF-A and its positive regulator HIF-1alpha through direct binding and stabilization of their mRNAs. This effect is mediated through the seeding sequence of the AUF1 protein in the *VEGF-A* and *HIF-1alpha* 3’UTR sequences. As a consequence, the expression of the 3 genes was highly correlative in various osteosarcoma cell lines, and AUF1 enhanced the pro-angiogenic capabilities of osteosarcoma cells both *in vitro* and *in vivo*. Indeed, while inhibition of AUF1 using specific siRNA suppressed the pro-angiogenic effects of osteosarcoma cells, ectopic expression of AUF1 enhanced the pro-angiogenic effect in a VEGF-A-dependent manner. Therefore, in the era of targeted therapy, anti-angiogenic therapies targeting AUF1 could provide effective methods for treating osteosarcoma.

## INTRODUCTION

Osteosarcoma is the most common primary bone malignancy in children and young adults. These aggressive tumors commonly metastasize and are highly resistant for the newly devised poly-chemotherapy regimens. Tumor angiogenesis, the growth of new blood vessels from pre-existing vasculature, is crucial for osteosarcoma growth, invasion and metastasis [1]. Indeed, therapies that block angiogenesis have shown clinical benefit in patients. However, the development of resistance to anti-angiogenic therapeutics and eventual tumor progression are very common, with unknown mechanism(s).

Vascular endothelial growth factor-A (VEGF-A), is a key angiogenic factor that has multiple functions, including vasculogenesis, inflammation, and vascular permeability, which are important in tumor angiogenesis [2]. Serum VEGF-A level is elevated in many cancer patients including osteosarcoma and has prognostic importance for osteosarcoma patients [3]. Inhibition of VEGF-A effectively suppresses angiogenesis in murine model of osteosarcoma [4]. However, the efficiency of anti-VEGF therapy in mouse models did not translate well to the clinic in humans, largely due to resistance to anti-VEGF therapy [5]. While VEGF-A expression in osteosarcoma has been associated with poor outcome, its regulatory mechanism(s) remains largely unknown.

The VEGF-A expression is regulated by hypoxia inducible factor-1 alpha (HIF-1α). HIF-1alpha is the first transcription factor response to hypoxia and is closely associated with angiogenesis. Under hypoxic conditions, HIF-1α accumulates in the cytoplasm, and then translocates into the nucleus to stimulate the transcription of a large number of genes including VEGF-A [6].

AUF1 (AU binding factor 1), also known as heterogenous nuclear ribonucleoprotein D (hnRNPD), has four isoforms (37, 40, 42, and 45 kDa), which result from alternative splicing of a single pre-mRNA [7]. These isoforms have various affinity for target transcripts, and p37 exhibits the strongest affinity [7, 8]. AUF1 binds various AU-rich conserved elements (ARE) in the 3’untranslated region (UTR) of several transcripts [9]. Although AUF1 is predominantly an mRNA-degrading protein [10–14], it is also involved in the stability and translation of several transcripts [15–17]. AUF1 target genes are involved in many physiological processes related to carcinogenesis, such as cell proliferation, apoptosis and transcription [9, 18, 19]. Indeed, AUF1 was found to be highly expressed in various cancers including breast, skin, thyroid and liver [18, 20]. Nonetheless, the role of AUF1 expression/activity in tumor initiation and/or progression still elusive.

Here, we present clear evidence that AUF1 is highly expressed in the aggressive osteosarcoma cell lines, and positively regulates the expression and secretion of the pro-angiogenic factor VEGF-A and its regulator HIF-1α. As a consequence, AUF1 enhances the pro-angiogenic capabilities of osteosarcoma cells *in vitro* and *in vivo*.

## RESULTS

### AUF1 positively regulates the expression of VEGF-A in osteosarcoma cells

**Figure 1:**
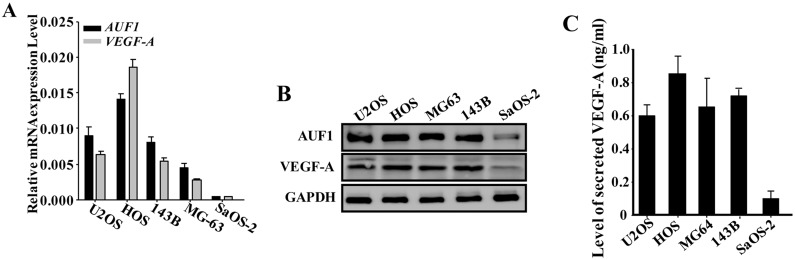
AUF1 positively regulates the expression of VEGF-A in osteosarcoma cells. **(A)** Total RNA was prepared from the indicated osteosarcoma cell lines and the levels of the *VEGF- A* and *AUF1* mRNAs were assessed by qRT-PCR. Error bars represent means ± SD of 3 different experiments. **(B)** Whole cell lysates were prepared from the indicated cells and used for immunoblotting analysis utilizing antibodies against the indicated proteins. **(C)** SFCM were collected from the indicated osteosarcoma cell lines after 24 h of culture and the level of the secreted VEGF-A was determined by ELISA. Error bars represent means ± SD of 3 different experiments.

To shed light on the role of AUF1 in the regulation of the pro-angiogenic capabilities of osteosarcoma cells, we first assessed the levels of the *AUF1* and *VEGF-A* mRNAs in various osteosarcoma cell lines by quantitative RT-PCR (qRT-PCR). Figure 1A shows that the mRNA levels of both *AUF1* and *VEGF-A* were highly correlative, both were higher in the highly aggressive and pro-metastatic osteosarcoma cell lines (U2OS, Human OsteoSarcoma HOS, MG63 and 143B) as compared to their levels in the less aggressive sarcoma osteogenic SaOS-2 cell line. Similar results were found at the protein levels (Figure 1B). This indicates the presence of positive correlation between AUF1 and VEGF-A in osteosarcoma cell lines. Next, serum-free conditioned media (SFCM) were collected from these cell lines and the level of the secreted VEGF-A was assessed by ELISA. Figure 1C shows that U2OS, HOS, MG63 and 143B cells secreted higher level of VEGF-A than SaOS-2 cells.

**Figure 2:**
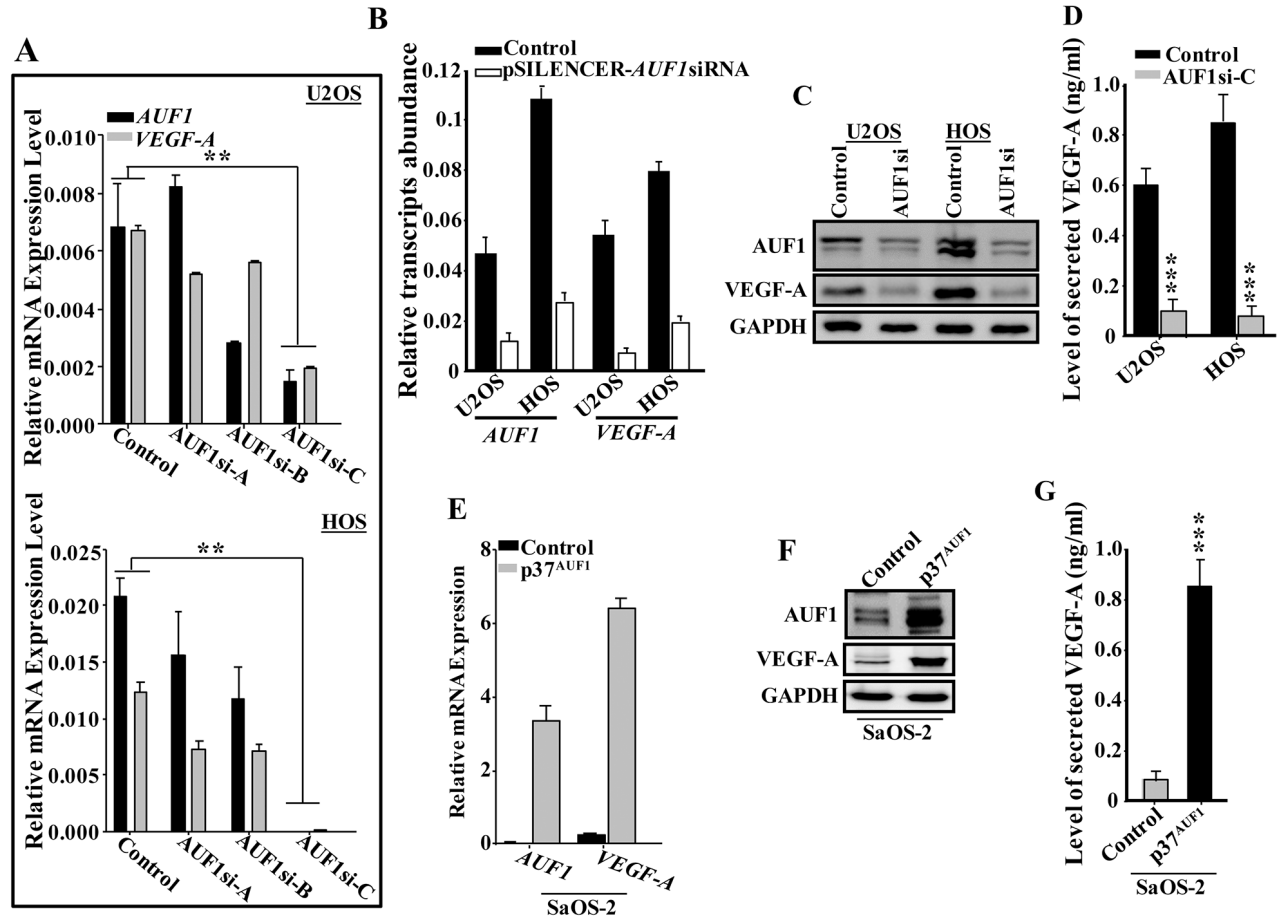
AUF1 positively controls the expression of VEGF-A. **(A** and **B)** U2OS and HOS cells were transfected with specific AUF1siRNA (3 different sequences) or pSILENCER- AUF1siRNA and scrambled sequences were used as controls. The generated cells (AUF1si-A, AUF1si-B, AUF1si-C and pSILENCER-AUF1siRNA) as well as their respective controls were used to prepare total RNA, which was then utilized to assess the levels of the *VEGF-A* and *AUF1* mRNAs by qRT-PCR. Error bars represents means ± SD. ^**^ *p* < 0.001. **(C)** Cell lysates were prepared from the indicated cells and were used for immunoblotting utilizing specific antibodies. (C) SFCM were collected from the indicated cells after 24 hrs of culture and the level of the secreted VEGF-A was determined by ELISA. Error bars represent means ± SD of 3 different experiments. ^***^ *p* < 0.00002. **(D)** p37^AUF1^ isoform was ectopically expressed in SaOS2 cells using empty vector as control. The generated cells (Control and p37^AUF1^) were utilized to prepare total RNA and the level of the indicated transcripts were assessed by qRT-PCR using specific primers. Error bars represent means ± SD. **(E)** whole cell lysates were prepared from the indicated cells and the levels of the indicated proteins were assessed by immunoblotting using specific antibodies. **(F)** SFCM were collected from the indicated cells after 24 hrs of culture and the level of the secreted VEGF-A was determined by ELISA. Error bars represent means ± SD of 3 different experiments. ^***^ *p* < 0.0000321.

To test the possible implication of AUF1 in the regulation of VEGF-A, AUF1 was downregulated in U2OS and HOS cells using specific siRNA (3 different sequences) and a scrambled sequence was used as control. The generated cells (AUF1si-A, AUF1si-B, AUF1si-C and control) were used to prepare total RNA and the levels of the *AUF1* and *VEGF-A* mRNAs were assessed by qRT-PCR. Figure 2A shows that the sequence C was the most efficient in down-regulating AUF1. Concomitantly, the level of the *VEGF-A* mRNA was also decreased, suggesting AUF1-dependent positive regulation of VEGF-A. Similar results were obtained using another siRNA that has been previously used [12, 21] (Figure 2B). Furthermore, these findings were confirmed at the protein level. Indeed, the immunoblot shows concomitant decrease of AUF1 and VEGF-A in AUF-1-deficient cells compared to control (Figure 2C).

Next, we assessed the level of secreted VEGF-A from AUF1si-C cells and their controls by ELISA. Figure 2D shows that the level of the secreted VEGF-A was also significantly reduced in the AUF1si-C cells as compared to control cells. Together, these data suggest that AUF1 positively controls the expression of VEGF-A. To confirm this, p37^AUF1^ isoform, which has the greatest affinity for the target transcript among other isoforms (5), was ectopically expressed in SaOS-2 cells, using an empty vector as control. The generated cells (Control and p37^AUF1^) were utilized to prepare whole cell lysates and the levels of the AUF1 and VEGF-A were analyzed by immunoblotting using specific antibodies. Figure 2E shows that upon AUF1 ectopic expression, the level of the *VAGF-A* mRNA was increased as compared to its level in the control cells. Similar result was found for the level of the VEGF-A protein upon ectopic expression of AUF1 in SaOS-2 cells (Figure 2F), as well as for the level of secreted VEGF-A (Figure 2G). These data further show that AUF1 positively regulates VEGF-A.

### AUF1 enhances the pro-angiogenic effects of osteosarcoma cells in a VEGF-A-dependent manner

**Figure 3:**
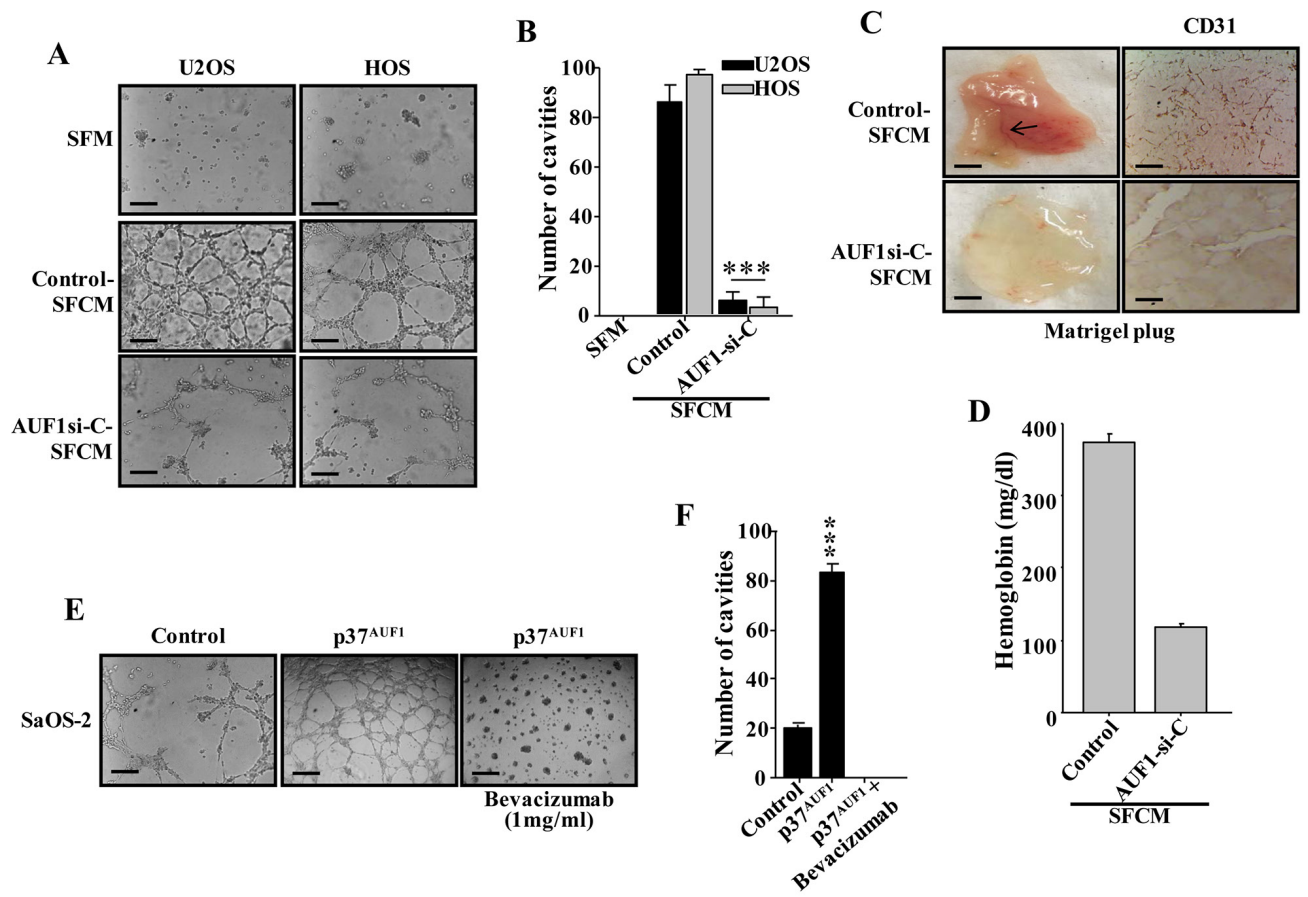
AUF1 enhances the capacity of osteosarcoma cells in promoting endothelial differentiation and angiogenesis in a VEGF-A-dependent manner. **(A** and **E)** SFCM were collected from the indicated cells and were applied independently on HUVEC cells plated on matrigel-coated 96-well plate, using SFM as negative control. The differentiation into capillary- like structures was assessed after 5 hrs of incubation. Representative photographs of HUVEC cavities are shown. Scale bars represent 30 *μ*m. **(B** and **F)** Histogram shows average number of microvessels observed in five different fields. Error bars represent means ± SD. ^***^ *P* ≤ 8.5×10^-7^. **(C)** *In-vivo* matrigel plug assay. SFCM were collected from 48 hrs cultures of the indicated cells, centrifuged and mixed with phenol-red-free matrigel, and were injected subcutaneously into nude mice. Mice were then sacrificed after 8 days and the matrigel plugs were extracted for staining with the CD31 antibody and for haemoglobin content **(D)**.

Next, we examined the role of AUF1 in osteosarcoma-dependent promotion of angiogenesis. To this end, serum-free medium (SFM) was conditioned for 48 hrs with AUF1-deficient U2OS and HOS cells or their control cells. The resulting SFCM were added separately to 96-well plate seeded with HUVEC cells (1×10^4^) in matrigel and used for *in vitro* angiogenic assay. SFM was also added as negative control. Figure 3A and 3B show that after 5 hrs of incubation the number of HUVEC cells that were differentiated into closed cavities was significantly higher in the presence of SFCM from U2OS and HOS cells compared to SFM. Interestingly, down-regulation of AUF1 significantly decreased the number of closed cavities (Figure 3A and 3B). This shows that AUF1 is an activator of the paracrine pro-angiogenic effects of U2OS and HOS cells.

Next, the same SFCM collected above were used to examine the paracrine effect of AUF1-deficient U2OS cells or control cells on vascular formation *in vivo*. To this end, SFCM was mixed with matrigel and injected subcutaneously into nude mice. Eight days later, the matrigel plugs were extracted and imaged for the vascular formation. Figure 3C shows that while SFCM from control cells promoted the vascular formation *in vivo*, down-regulation of AUF1 clearly suppressed the formation of blood vessels. Next, the level of CD31, as an endothelial cell marker, was assessed in the matrigel plugs by immunohistochemistry using anti-CD31 antibody. Figure 3C shows very low density of CD31-positive cells in matrigel plugs containing SFCM from AUF1-deficient U2OS cells as compared to SFCM from control cells. In addition, we assessed the haemoglobin level, a surrogate for functional blood flow, in the matrigel plugs and showed that down-regulation of AUF1 in U2OS cells significantly reduced the concentration of haemoglobin (Figure 3D). This shows the pro-angiogenic role of AUF1 in *vivo* as well.

Next, SFCM were collected from p37^AUF1^-expressing SaOS-2 cells and their control counterparts, and were added separately to HUVEC cells (1×10^4^) in matrigel and used for *in vitro* angiogenic assay. SFM was also added as negative control. Figure 3E and 3F show that after 5 hrs of incubation the number of HUVECs that were differentiated into closed cavities was significantly higher in the presence of SFCM from p37^AUF1^-expressing SaOS-2 cells as compared to the SFCM from their control counterpart cells. Interestingly, specific inhibition of VEGF-A in the SFCM from p37^AUF1^-expressing SaOS-2 cells by Bevacizumab significantly inhibited the formation of the closed cavities (Figure 3E and 3F). Collectively, these data show that AUF1 enhances the pro-angiogenic capabilities of osteosarcoma cells in a VEGF-A-dependent manner.

### AUF1 binds and stabilizes the *VEGF-A* mRNA

**Figure 4:**
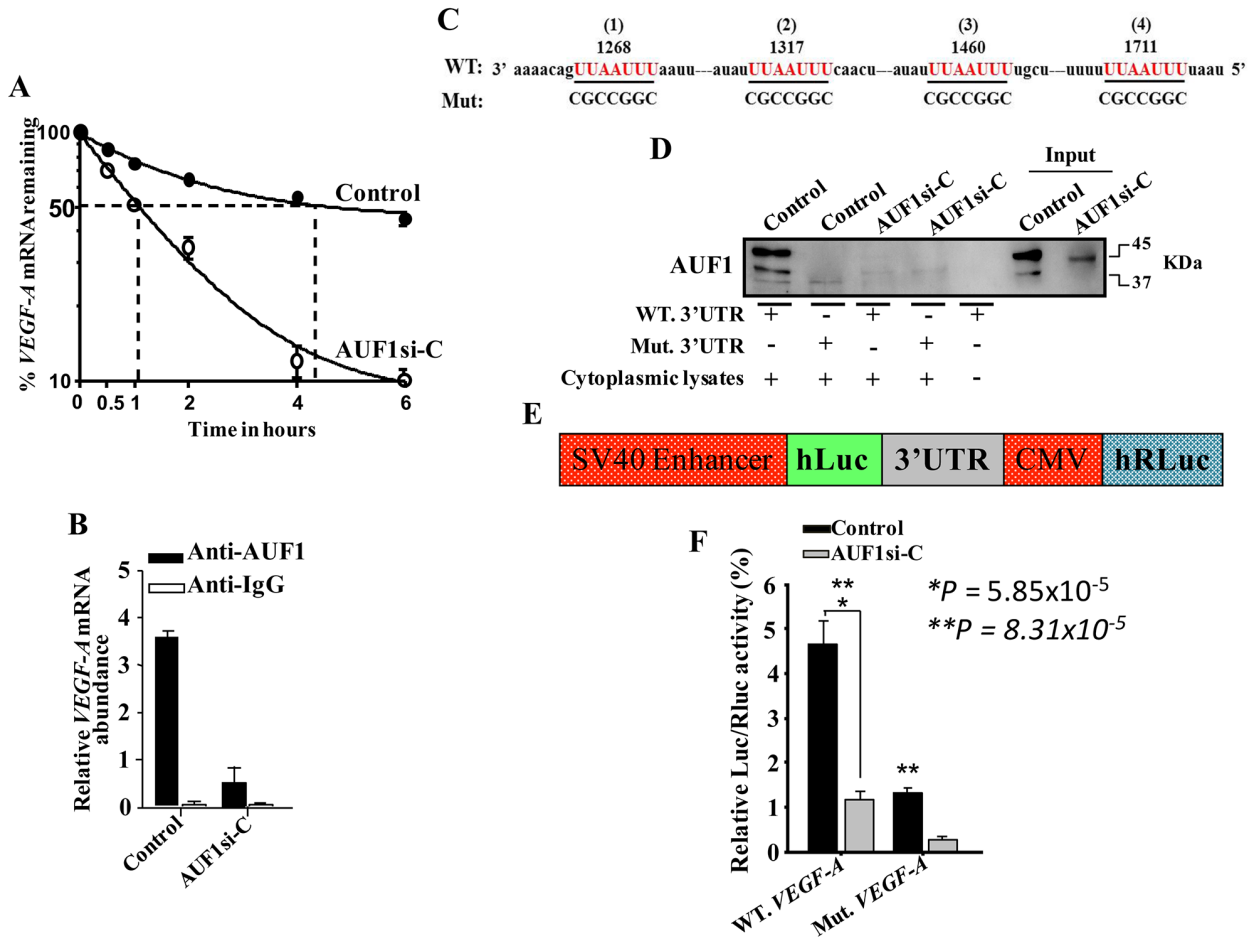
AUF1 binds to and stabilizes the *VEGF-A* mRNA. **(A)** AUF1-Deficient U2OS cells and their control counterparts were treated with actinomycin D, and then re-incubated for the indicated periods of time. Total RNA was extracted and the amount of the *VEGF-A* mRNA was assessed using qRT-PCR. The graph shows the proportion of the *VEGF-A* mRNA remaining post-treatment, and the dotted lines indicate the *VEGF-A* mRNA half-life. Error bars represent means ± SD of 3 different experiments. **(B)** RNAs bound to the AUF1 protein were isolated by immunoprecipitation from AUF1-defecient U2OS cells and their control counterparts using anti- AUF1 antibody or anti-IgG (Control), and then the *VEGF-A* mRNA was amplified by qRT-PCR. Error bars represent means ± SD of 3 different experiments. **(C)** Sequence alignment of the human AUF1 binding sites in the *VEGF-A* 3’UTR. **(D)** Biotinylated *VEGF-A* 3’UTR bearing either wild type or mutated sequence of the second AUF1 binding site was incubated with cytoplasmic cellular lysate from the indicated cells and the association of AUF1 with these RNAs was detected by immunoblotting using anti-AUF1 antibody. **(E)** Schematic representation of the luciferase reporter vector bearing the *VEGF-A* 3’UTR. **(F)** AUF1-deficient U2OS cells and their control counterparts were stably transfected with the luciferase reporter vector bearing wild- type *VEGF-A* 3’UTR or a mutated sequence for the binding site of AUF1. The reporter activity was assessed at 48 hrs post-transfection. Data (Mean ±SEM, n = 4) were presented as % change in reporter activity as compared to the negative control cells (*) or to the wild-type 3’UTR (^**^). ^*^ and ^**^ *P* <0.000031.

Next, we investigated the possible AUF1- dependent stabilization of the *VEGF-A* mRNA. Thereby, AUF1-defecient U2OS cells and their control counterparts were treated with actinomycin D, the transcription inhibitor, and then re- incubated for different periods of time (0-6 hrs). Total RNA was prepared and the mRNA level of *VEGF-A* was measured by qRT-PCR. Figure 4A indicates that the down-regulation of AUF1 in U2OS cells significantly reduced the *VEGF-A* mRNA half-life. Indeed, while the *VEGF-A* mRNA half-life was 4 hrs 10 min in control cells, it was reduced to only 1 hr in AUF1- deficient U2OS cells (Figure 4A). This indicates that AUF1 increases the stability of the *VEGF-A* mRNA. To confirm this, we studied the binding of AUF1 to the *VEGF-A* mRNA. To this end, whole cell lysates were prepared from AUF1-deficient U2OS cells and their control counterparts, and the AUF1-mRNAs ribonucleoprotein complexes were obtained by immunoprecipitation (IP) utilizing anti-AUF1 antibody or anti-IgG (Control), and then the *VEGF-A* mRNA was amplified by qRT-PCR. The *VEGF-A* mRNA was amplified showing the binding of the AUF1 protein to this transcript (Figure 4B). The level of the *VEGF-A* transcript that was associated with AUF1 was also reduced in AUF1-deficient U2OS cells as compared to the control cells (Figure 4B). These findings indicate that AUF1 binds and decreases the turnover of the *VEGF-A* mRNA.

### AUF1 controls the VEGF-A mRNA expression via its 3’UTR

To shed more light on the binding of AUF1 to the *VEGF-A* 3’UTR, we first explored for AUF1 binding site(s) on the 3’UTR of the *VEGF-A* mRNA, and we have found 4 different AUF1 binding sites (Figure 4C). Thereby, we examined the implication of these binding sites in the binding of AUF1 to the *VEGF-A* mRNA. Therefore, we synthesized biotinylated *VEGF-A* 3’UTR spanning either wild-type or mutated AUF1 binding site, and then were incubated with cytoplasmic cellular lysates prepared from AUF1-deficient U2OS cells or their control counterparts. The 3’UTR/AUF1 ribonucleoprotein complexes were immuoprecipitated and AUF1 level was assessed by immunoblotting. AUF1 was associated with the wild-type *VEGF-A* 3’UTR in control cells, however this association was significantly reduced when AUF1 was knocked-down in U2OS cells. Intriguingly, the AUF1 associating to the *VEGF-A* 3’UTR was abolished when the corresponding sites were mutated. This result indicates the binding of the AUF1 protein to the *VEGF-A* 3’UTR *in vitro*. We next investigated the potential role of the AUF1 binding sites in the *VEGF-A* 3’UTR in the control of VEGF-A expression. Therefore, wild-type *VEGF-A* 3’UTR or mutated sequence for AUF1 binding site were integrated into a luciferase/Renilla reporter vector (Figure 4E) and were introduced into AUF1-deficient U2OS cells or their control counterparts. The reporter activity fused to the wild-type sequence of the *VEGF-A* 3’UTR was significantly reduced in AUF1-deficient U2OS cells as compared to controls (Figure 4F), and this effect was eliminated by mutating the presumed AUF1 binding sites within the 3’UTR of the *VEGF-A* mRNA (Figure 4F). This shows that the AUF1 effect is mediated through association with its seeding sequence in the 3’UTR of the *VEGF-A* transcript.

### AUF1 positively regulates the expression of HIF-1α in osteosarcoma cell lines

**Figure 5:**
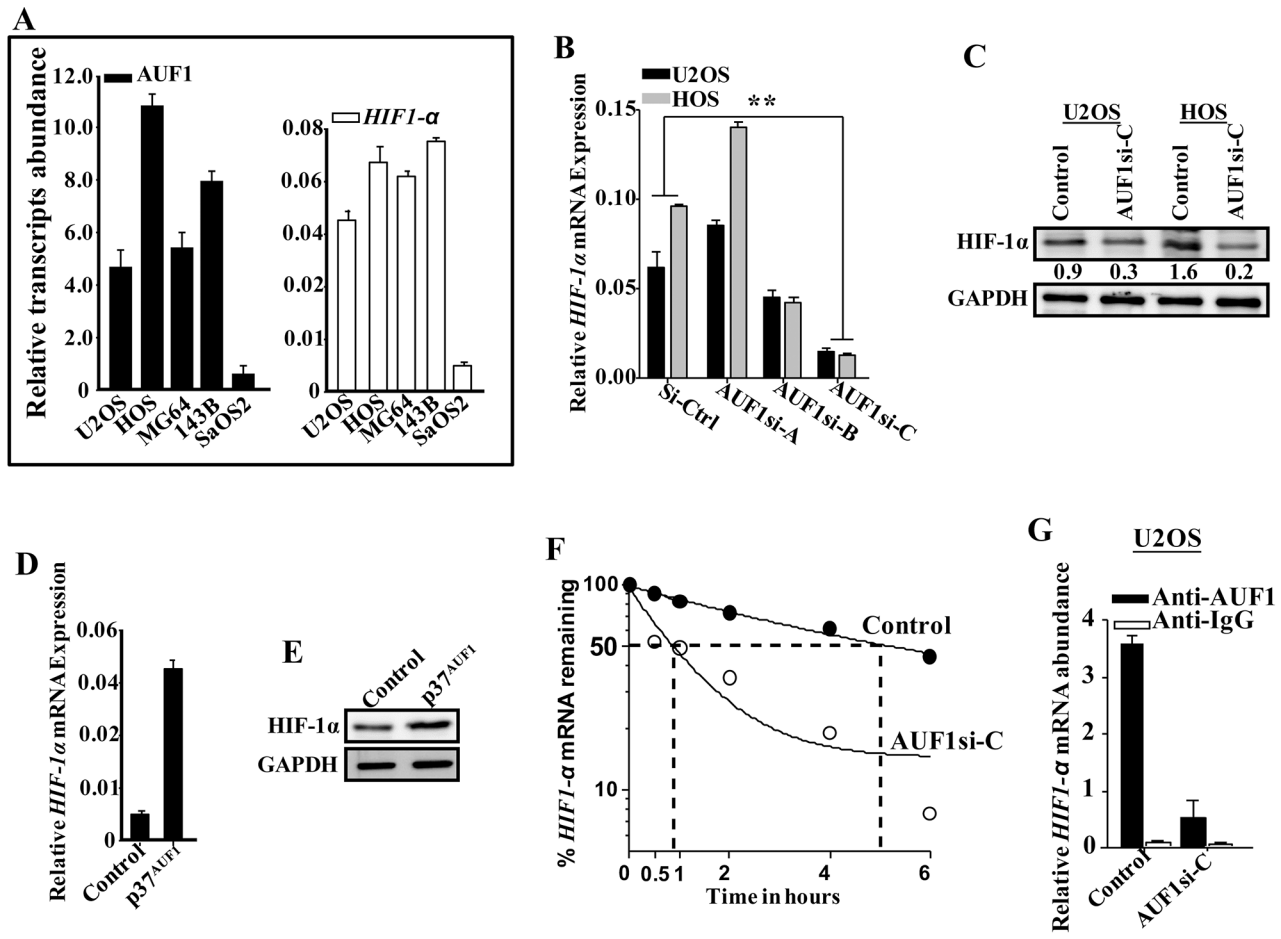
AUF1 stabilizes the *HIF-1α* mRNA. **(A)** Total RNA was prepared from the indicated osteosarcoma cell lines and the levels of the *HIF-1α* and *AUF1* mRNAs were assessed by qRT- PCR. Error bars represent means ± SD of 3 different experiments. **(B)** Total RNA was prepared from AUF1-deficient U2OS and HOS cells and their control counterparts, and the level of the *HIF-1α* mRNA was assessed by qRT-PCR. Error bars represent means ± SD of 3 different experiments. **(C-E)** Figure legends are as in Figure 2B and 2D–2E, respectively. **(F** and **G)** Figure legends are as in Figure 4A and 4B, respectively.

Since HIF-1α positively controls VEGF-A, we investigated the possible involvement of AUF1 in the regulation of HIF-1α as well. Therefore, total RNA was prepared from osteosarcoma cell lines and the levels of *AUF1* and *HIF-1α* mRNAs were measured by qRT-PCR. Figure 5A indicates that the levels of both mRNAs were higher in the highly aggressive and pro- metastatic osteosarcoma cell lines (U2OS, HOS, MG63 and 143B) as compared to their levels in the less aggressive SaOS-2 cell line. To confirm the role of AUF1 in the control of HIF-1α, the level of the *HIF-1α* mRNA was measured in the AUF1-deficient U2OS and HOS cells by qRT- PCR. Figure 5B shows that the AUF1-siRNA-C, which strongly down-regulated AUF-1 (Figure 2A), was the most efficient in down-regulating HIF-1α at both mRNA and protein levels as compared to controls (Figure 5B and 5C). Furthermore, upon ectopic expression of AUF1 in SaOS-2 cells, the level of the *HIF-1α* mRNA was increased as compared to its level in the control cells (Figure 5D). Similar result was found for the level of the HIF-1α protein upon ectopic expression of AUF1 in SaOS-2 cells (Figure 5E). These data clearly show that AUF1 positively regulates HIF-1α.

### AUF1 binds and stabilizes the *HIF-1α* mRNA

Next, we investigated the possible AUF1-dependent stabilization of the *HIF-1α* mRNA. Thereby, AUF1-deficient U2OS cells and their control counterparts were exposed to actinomycin D, and then re-incubated for different periods of time (0-6 hrs). Total RNA was prepared and the *HIF-1α* mRNA level was measured by qRT-PCR. Figure 5F indicates that AUF1 down-regulation in U2OS cells significantly reduced the *HIF-1α* mRNA half-life. Indeed, while the *HIF-1α* mRNA half-life was 5 hrs in control cells, it was reduced to less than 1 hr in AUF1-deficient U2OS cells (Figure 5F). This indicates that AUF1 enhances the stability of the *HIF-1α* mRNA.

We next studied the binding of AUF1 to the *HIF-1α* mRNA. To do this, whole cell lysates were prepared from AUF1-deficient U2OS cells or their control counterparts, and the AUF1-mRNAs ribonucleoprotein complexes were obtained by immunoprecipitation (IP) using anti-AUF1 or anti-IgG antibodies, and then qRT- PCR was used for the amplification of the *HIF-1α* mRNA. Figure 5G shows amplification of the *HIF-1α* mRNA, showing the association of AUF1 with the *HIF-1α* mRNA.

AUF1 with the *HIF-1α* mRNA. Notably, the level of the *HIF-1α* mRNA that was associated to AUF1 was reduced in AUF1-deficient U2OS cells as compared to control cells (Figure 5D). These data indicate that AUF1 binds and stabilizes the *HIF-1α* mRNA.

### AUF1 controls the *HIF-1α* mRNA level via its 3’UTR

**Figure 6:**
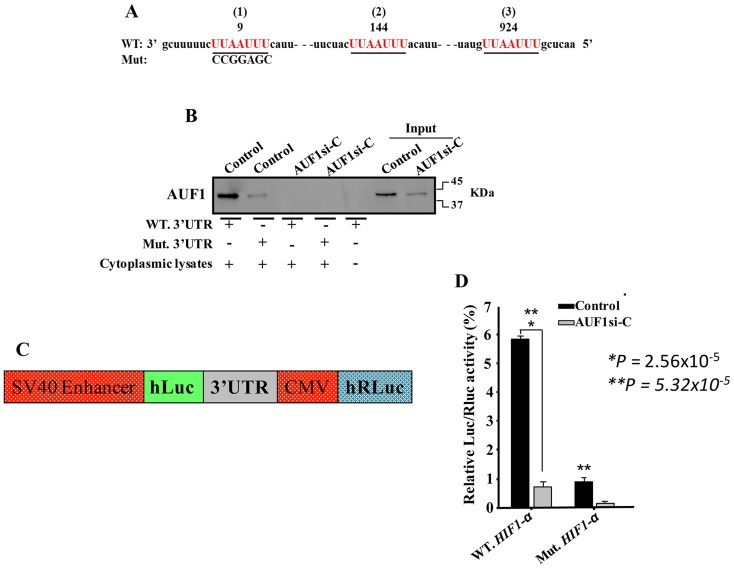
AUF1 binds to the *HIF-1α* 3’UTR. **(A-D)** Figure legends are as in Figure 4C–4F, respectively.

Three different AUF1 binding sites were found on the 3’UTR of the *HIF-1α* mRNA (Figure 6A). Thereby, we tested the association of AUF1 with the *HIF-1α* mRNA 3’UTR. Biotinylated *HIF-1α* 3’UTR spanning either wild-type or mutated AUF1 binding site were synthesized and incubated with cytoplasmic cellular extracts purified from AUF1-deficient U2OS cells and their control counterparts. The 3’UTR/AUF1 ribonucleoprotein complexes were immunoprecipitated and the AUF1 protein level was assessed. Figure 6B shows that AUF1 was associated with the *HIF-1α* 3’UTR in control cells, and this association was reduced when AUF1 was knocked-down in U2OS cells. Interestingly, mutated AUF1 binding site abolished its binding to the *HIF-1α* 3’UTR. This result indicates the binding of AUF1 to the *HIF-1α* 3’UTR *in vitro*. To corroborate this, we investigated the possible involvement of the AUF1 binding sites in the *HIF-1α* mRNA 3’UTR on the control of HIF-1α expression. Therefore, intact *HIF-1α* 3’UTR or mutated sequence for AUF1 binding sites were integrated into a luciferase/Renilla reporter vector (Figure 6C) and were introduced into AUF1-deficient U2OS cells or their control counterparts. The reporter activity fused to the intact sequence of the *HIF-1α* 3’UTR was significantly reduced in AUF1-deficient U2OS cells as compared to controls (Figure 6D). This effect was eliminated by mutating the presumed AUF1 binding site within the 3’UTR of the *HIF-1α* mRNA (Figure 6D). This indicates that the effect of AUF1 is mediated via association with its seeding sequence in the *HIF-1α* 3’UTR.

## DISCUSSION

Angiogenesis is a major hallmark of cancer, and plays key roles in the growth and spread of various types of tumors, including osteosarcoma. Thereby, the identification of the genes and pathways that control this essential pro-metastatic process is of great importance in order to develop more efficient and precise therapeutics for these deadly diseases. In the present study, we have shown that the expression of the pro-angiogenic factor VEGF-A is under the control of the RNA binding protein AUF1. Indeed, while specific down-regulation of AUF1 decreased the expression/secretion of VEGF-A, ectopic expression of AUF1 up-regulated VEGF-A. Mechanistically, we have shown direct binding of AUF1 to the *VEGF-A* 3’UTR and stabilization of its mRNA. This was corroborated by showing that mutated AUF1 binding sites in the *VEGF- A* 3’UTR abolishes its regulatory effects on VEGF-A. Importantly, while down-regulation of AUF1 abolished the pro-angiogenic potential of osteosarcoma cells, ectopic expression of AUF1 enhanced this capacity in a VEGF-A-dependent manner. This provided clear evidence that AUF1 is an important player in osteosarcoma angiogenesis through positive regulation of VEGF-A. VEGF-A, the most effective angiogenic molecule, is usually highly expressed in osteosarcoma. Furthermore, circulating VEGF level was associated with lung metastasis, and significant positive correlation was observed between VEGF levels and osteosarcoma tumor stages [2]. Interestingly, like VEGF-A, AUF1 was also upregulated in highly aggressive osteosarcoma cell lines. Moreover, AUF1 has been shown to bind to transcripts encoding immune regulators such as the interleukins IL-1β, IL-2, IL-3, IL-6, TNF-α and many other mRNAs, which might indirectly affect angiogenesis [10]. Together, these studies implicate AUF1 in the regulation of the angiogenesis process through controlling the expression of several pro-angiogenic molecules.

**Figure 7:**
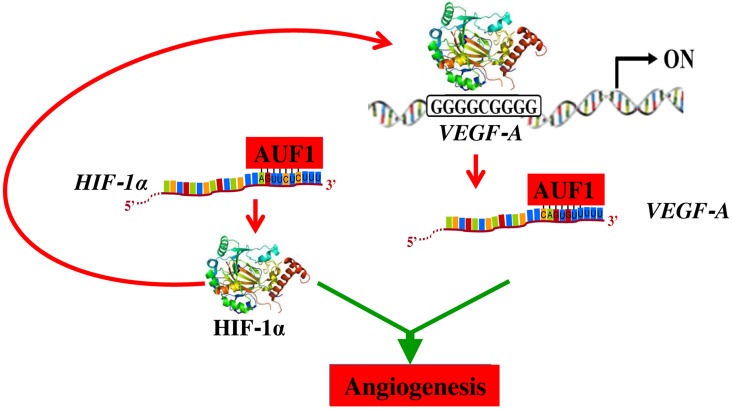
Schematic representation of the role of AUF1 in the stabilization of the VEGF-A and *HIF-1alpha* mRNAs and implication in angiogenesis. See text for more details.

In response to hypoxia, tumor tissues produce and secrete high levels of several pro- angiogenenic factors such as VEGF-A, which transcription is under the control of the hypoxia- inducible factor HIF-1α. This transcription factor is a major inducer of blood vessel growth during tumorigenesis, through regulating the expression of a plethora of pro-angiogenic genes [22]. Importantly, we have shown here that, like VEGF-A, HIF-1α is also a target of AUF1, which binds and stabilizes the *HIF-1α* mRNA. This indicates that AUF1 may also promote angiogenesis through indirect upregulation of VEGF-A via induction of its direct transcription factor HIF-1α (Figure 7). Like AUF1 and VEGF-A, HIF-1α was also highly expressed in the most aggressive osteosarcoma cells (Figure 5A). Recently, Tsai et al have shown the implication of HIF-1α in the promotion of osteosarcoma angiogenesis [23]. Furthermore, siRNA mediated specific inhibition of HIF-1α inhibited angiogenesis in osteosarcoma [24]. These results indicate that HIF-1α is an important regulator of angiogenesis in osteosarcoma, and therefore its inhibition could constitute an attractive therapeutic strategy for these neoplasms.

In summary, the present findings present clear evidence that AUF1 is a master regulator of osteosarcoma angiogenesis through positive regulation of 2 key angiogenic factors VEGF-A and its transcriptional regulator HIF-1α. This also indicates that AUF1 controls the expression of VEGF-A both directly through binding and stabilizing its mRNA, and indirectly via HIF-1α upregulation (Figure 7). In the era of precision medicine and targeted therapy, inhibition of angiogenesis through specific targeting of AUF1 could constitute an efficient therapeutic approach for osteosarcoma. In addition, the level of AUF1 could be of enormous prognostic/diagnostic values for osteosarcoma patients.

## MATERIALS AND METHODS

### Cell lines, cell culture and reagents

Osteosarcoma cell lines (U2OS, HOS, MG63, 143B and SaOS-2) were purchased from ATCC (Manassas, VA) and were cultured following the instructions of the company. All supplements were purchased from Gibco (Grand Island, NY). Cells were maintained at 5% CO_2_ and 37°C humidified incubator. Actinomycin D was purchased from Sigma-Aldrich (St. Louis, USA).

### AUF1 binding sites prediction

AUF1 binding sites on the 3’UTR of its targets were identified utilizing the algorithms miRanda Human miRNA targets.

### RNA preparation and quantitative RT-PCR

Total RNA was prepared using the mRNeasy mini kit (Qiagen, UK) according to the manufacturer's instructions and was treated with RNase-free DNase before cDNA synthesis using Advantage RT for PCR kit (Clontech Laboratories, UK). Quantitative RT-PCR was performed using FastStart Essential DNA master (Roche) and the amplifications were performed utilizing the 96 Real time PCR detection system (Roche). The melting-curve data were collected to check PCR specificity, and the amount of PCR products was measured by threshold cycle (Ct) values and the relative ratio of specific genes to *GAPDH* for each sample was then calculated. The utilized primers were:

*AUF1:* 5’-GATCAAGGGGTTTTGGCTTT-3’ and 5’-GTTGTCCATGGGGACCTCTA-3’

*VEGF-A* 5’-CCCACTGAGGAGTCCAACAT-3’ and 5’-TTTCTTGCGCTTTCGTTTTT-3’

*HIF-1α*: 5’-TCATCAGTTGCCACTTCCCCA-3’ and 5’-CCGTCATCTGTTAGCACCATCAC-3’

*GAPDH*: 5’-GAGTCCACTGGCGTCTTC-3’ and 5’-GGGGTGCTAAGCAGTTGGT-3’

### Immunoprecipitation and RT-PCR

Cell extracts were prepared from confluent cells, and 3 mg of proteins were incubated in the lysis buffer (50 mM Tris (pH 8), 100 mM NaCl, 10% glycerol, protease inhibitors, 5 mM DTT and 2 U/ml RNasin) and 5 μg of AUF1 mouse monoclonal antibody (mouse IgG1 was used as control) were added and mixed at 4°C for 4 hrs. Equal volume of protein A agarose was added per immunoprecipitation and mixed overnight at 4°C. After centrifugation, the pellet was re- suspended in 1 ml TRI reagent used for RNA extraction. RT–PCR reactions were performed as described above.

### Transfection and viral infection

*AUF1*siRNA and control siRNA were obtained from Origene Technologies (Rockville, MD, USA). siRNA Sequence: (rGrCrCrArUrGrUrCrGrArArGrGrArArCrArArUrArUrCrAGC, and universal sequence was used as a negative control). In addition, pSILENCER-AUF1siRNA and control-siRNA plasmids [21] were utilized. The transfections were carried out using the High Perfect reagent (Qiagen), as recommended by the manufacturer. Cells were incubated for 3 days after transfection, recovered and then were re-cultured for 3 days before collection for subsequent experiments. pLenti-GIII-CMV-hHNRNPD-GFP-2A-Puro (Expressing the p37^AUF1^ isoform) (Applied Biological Materials Inc.) and their control plasmids were used at 1 µg/ml each for transfection of 293FT cells. Lentiviral supernatants were collected 48 h post- transfection. Culture media were removed from the target cells and replaced with the lentiviral supernatant and incubated for 24 hrs in presence of 1 µg/ml polybrene (Sigma-Aldrich). Transduced cells were selected after 48 hrs with puromycin or G418.

### Dual-luciferase reporter assay

U2OS cells were plated at 1×10^5^ cells/well on 6-well plates and transfected with 3 µg of the luciferase/Renilla reporter vector containing either human full *VEGF-A* 3’UTR (871 bP), mutated sequence of the AUF1 seed sequence or a control sequence containing no-ARE sequence of *VEGFA* 3’UTR (GeneCopoeia). Transfection was carried out using Lipofectamin 2000 as recommended by the manufacturer (Invitrogen). At 24 hrs post-transfection, cells were seeded in 96-well plate and Firefly and Renilla luciferase activities were consecutively measured using the dual-luciferase assay as recommended by the manufacturer (GeneCopoeia). The Firefly luciferase signal was normalized to the Renilla luciferase signal for each individual analysis. The mean and SEM were calculated from three wells for each 3’UTR activity and presented as fold change over the non-stimulated control.

### Biotin pull-down analysis

The probes used to prepare biotinylated transcripts spanning the *VEGFA* 3’UTR are: (Wild type) UAAUUAGAAAUUAAAACAGUUAAUUUAAUUAAAGAGUAGGGUUU and (Mutated) UAAUUAGAAAUUAAAACAGCCGCUAUAAUUAAAGAGUAGGGUUU. *HIF-1α* 3’UTR are: (Wild type) GCUUUUUCUUAAUUUCAUUCCUUUUUUUGGACACUGGUGGC and (Mutated) GCUUUUUCCCAGCGGCAUUCCUUUUUUUGGACACUGGUGGC. Biotinylation was performed using the RNA 3’ End Biotinylation kit as instructed by the manufacturer (Thermo Scientific, USA). Cytoplasmic lysates (200 µg per sample) were incubated with 3 µg of purified biotinylated transcripts for 30 min at room temperature, and then the complexes were precipitated with streptavidin-coupled Dynabeads (Invitrogen, USA) as previously described [25]. Proteins present in the pull-down material were analyzed by immunoblot analysis.

### Cellular lysate preparation and immunoblotting

This has been performed as previously described (5). Antibodies directed against AUF1 and VEGF-A (VG-1) were purchased from Abcam. HIF-1α (H1α67) and GAPDH (14C10) from Cell Signaling Technology.

### Analysis of mRNA stability

Cells were challenged with Actinomycin D (5 µg/ml) for various periods of time (0-6 hrs), and then total RNA was purified and assessed using qRT-PCR. Nonlinear regression analysis One-phase exponential decay curve analysis (GraphPad Prism) (GraphPad software 5.03, Inc) was used to assess mRNA decay kinetics [26].

### HUVEC endothelial tube formation assay

The formation of capillary-like structures was assessed in a 96-well plate coated with ice- cold growth factor-reduced Matrigel (*in vitro* angiogenesis assay, Millipore). After solidification of the matrix at 37ºC, 1×10^4^ HUVEC cells were seeded onto the polymerized matrix in the presence of 200 µl of conditioned medium. Formation of capillary-like structure was photographed after 5 hrs of incubation and their number was counted. The total tube area was obtained from five random microscopic fields and expressed as a mean of three different experiments.

### *In-vivo* matrigel plug assay

Serum-free conditioned media (SFCM) were collected from 48 hrs cultures and centrifuges to remove cells. SFCM was then mixed with phenol-red-free matrigel (2:3 proportion, total 0.5 ml; BD Biosciences). Subcutaneous (s.c.) injection in mice was performed and the mice were killed after 8 days and the matrigel plugs were extracted for haemoglobin content (QuantiChromhaemoglobin assay; BioAssay Systems), and immunohistochemistry for CD31 antibody.

### Enzyme-Linked Immunosorbent Assay (ELISA)

Conditioned media from 48 hrs cell cultures were harvested, and ELISA was performed according to the manufacturer's instructions (R&D Systems, Minneapolis, MN). The OD was used at 450 nm on x-mark microplate reader (Bio-Rad).

### Statistical analysis

Statistical analysis was performed using student’s *t*-test and *P* values of 0.05 and less were considered as statistically significant.

## References

[1] Gorlick R , Anderson P , Andrulis I , Arndt C , Beardsley GP , Bernstein M , Bridge J , Cheung NK , Dome JS , Ebb D , Gardner T , Gebhardt M , Grier H , et al. Biology of childhood osteogenic sarcoma and potential targets for therapeutic development: meeting summary. Clin Cancer Res. 2003; 9:5442–5453. 14654523

[2] Xie L , Ji T , Guo W . Anti-angiogenesis target therapy for advanced osteosarcoma (Review). Oncol Rep. 2017; 38:625–636. 10.3892/or.2017.5735. 28656259 PMC5562076

[3] DuBois S , Demetri G . Markers of angiogenesis and clinical features in patients with sarcoma. Cancer. 2007; 109:813–819. 10.1002/cncr.22455. 17265525

[4] Zhao J , Zhang ZR , Zhao N , Ma BA , Fan QY . VEGF silencing inhibits human osteosarcoma angiogenesis and promotes cell apoptosis via PI3K/AKT signaling pathway. Int J Clin Exp Med. 2015; 8:12411–12417. 10.1007/s12013-015-0692-7. 26550152 PMC4612837

[5] Sitohy B , Nagy JA , Dvorak HF . Anti-VEGF/VEGFR therapy for cancer: reassessing the target. Cancer Res. 2012; 72:1909–1914. 10.1158/0008-5472.CAN-11-3406. 22508695 PMC3335750

[6] Semenza GL . Defining the role of hypoxia-inducible factor 1 in cancer biology and therapeutics. Oncogene. 2010; 29:625–634. 10.1038/onc.2009.441. 19946328 PMC2969168

[7] Wagner BJ , DeMaria CT , Sun Y , Wilson GM , Brewer G . Structure and genomic organization of the human AUF1 gene: alternative pre-mRNA splicing generates four protein isoforms. Genomics. 1998; 48:195–202. 10.1006/geno.1997.5142. 9521873

[8] Kajita Y , Nakayama J , Aizawa M , Ishikawa F . The UUAG-specific RNA binding protein, heterogeneous nuclear ribonucleoprotein D0. Common modular structure and binding properties of the 2xRBD-Gly family. J Biol Chem. 1995; 270:22167–22175. 10.1074/jbc.270.38.22167. 7673195

[9] White EJ , Brewer G , Wilson GM . Post-transcriptional control of gene expression by AUF1: mechanisms, physiological targets, and regulation. Biochim Biophys Acta. 2013; 1829:680–688. 10.1016/j.bbagrm.2012.12.002. 23246978 PMC3664190

[10] Gratacos FM , Brewer G . The role of AUF1 in regulated mRNA decay. Wiley Interdiscip Rev RNA. 2010; 1:457–473. 10.1002/wrna.26. 21956942 PMC3608466

[11] Lal A , Mazan-Mamczarz K , Kawai T , Yang X , Martindale JL , Gorospe M . Concurrent versus individual binding of HuR and AUF1 to common labile target mRNAs. EMBO J. 2004; 23:3092–3102. 10.1038/sj.emboj.7600305. 15257295 PMC514922

[12] Al-Khalaf HH , Aboussekhra A . p16(INK4A) Positively Regulates p21(WAF1) Expression by suppressing AUF1-dependent mRNA decay. PLoS One. 2013; 8:e70133. 10.1371/journal.pone.0070133. 23894605 PMC3720951

[13] Al-Khalaf HH , Colak D , Al-Saif M , Al-Bakheet A , Hendrayani SF , Al-Yousef N , Kaya N , Khabar KS , Aboussekhra A . p16(INK4a) positively regulates cyclin D1 and E2F1 through negative control of AUF1. PLoS One. 2011; 6:e21111. 10.1371/journal.pone.0021111. 21799732 PMC3140473

[14] Brewer G , Saccani S , Sarkar S , Lewis A , Pestka S . Increased interleukin-10 mRNA stability in melanoma cells is associated with decreased levels of A + U-rich element binding factor AUF1. J Interferon Cytokine Res. 2003; 23:553–564. 10.1089/107999003322485053. 14585195

[15] Liao B , Hu Y , Brewer G . Competitive binding of AUF1 and TIAR to MYC mRNA controls its translation. Nat Struct Mol Biol. 2007; 14:511–518. 10.1038/nsmb1249. 17486099

[16] Sarkar S , Sinsimer KS , Foster RL , Brewer G , Pestka S . AUF1 isoform-specific regulation of anti-inflammatory IL10 expression in monocytes. J Interferon Cytokine Res. 2008; 28:679–691. 10.1089/jir.2008.0028. 18844578 PMC2956575

[17] Palanisamy V , Park NJ , Wang J , Wong DT . AUF1 and HuR proteins stabilize interleukin-8 mRNA in human saliva. J Dent Res. 2008; 87:772–776. 10.1177/154405910808700803. 18650551 PMC2572714

[18] Zucconi BE , Wilson GM . Modulation of neoplastic gene regulatory pathways by the RNA- binding factor AUF1. Front Biosci (Landmark Ed). 2011; 16:2307–2325. 10.2741/3855. 21622178 PMC3589912

[19] Arcondeguy T , Lacazette E , Millevoi S , Prats H , Touriol C . VEGF-A mRNA processing, stability and translation: a paradigm for intricate regulation of gene expression at the post- transcriptional level. Nucleic Acids Res. 2013; 41:7997–8010. 10.1093/nar/gkt539. 23851566 PMC3783158

[20] Abdelmohsen K , Tominaga-Yamanaka K , Srikantan S , Yoon JH , Kang MJ , Gorospe M . RNA-binding protein AUF1 represses Dicer expression. Nucleic Acids Res. 2012; 40:11531–11544. 10.1093/nar/gks930. 23066106 PMC3526313

[21] Wang W , Martindale JL , Yang X , Chrest FJ , Gorospe M . Increased stability of the p16 mRNA with replicative senescence. EMBO Rep. 2005; 6:158–164. 10.1038/sj.embor.7400346. 15678155 PMC1299256

[22] Li YS , Liu Q , Tian J , He HB , Luo W . Angiogenesis Process in Osteosarcoma: An Updated Perspective of Pathophysiology and Therapeutics. Am J Med Sci. 2019; 357:280-288. 10.1016/j.amjms.2018.12.004. 30711189

[23] Tsai HC , Tzeng HE , Huang CY , Huang YL , Tsai CH , Wang SW , Wang PC , Chang AC , Fong YC , Tang CH . WISP-1 positively regulates angiogenesis by controlling VEGF-A expression in human osteosarcoma. Cell Death Dis. 2017; 8:e2750. 10.1038/cddis.2016.421. 28406476 PMC5477571

[24] Zhang XD , Wu Q , Yang SH . Effects of siRNA-mediated HIF-1alpha gene silencing on angiogenesis in osteosarcoma. Pak J Med Sci. 2017; 33:341–346. 10.12669/pjms.332.12587. 28523034 PMC5432701

[25] Tominaga K , Srikantan S , Lee EK , Subaran SS , Martindale JL , Abdelmohsen K , Gorospe M . Competitive regulation of nucleolin expression by HuR and miR-494. Mol Cell Biol. 2011; 31:4219–4231. 10.1128/MCB.05955-11. 21859890 PMC3187287

[26] Al-Haj L , Blackshear PJ , Khabar KS . Regulation of p21/CIP1/WAF-1 mediated cell-cycle arrest by RNase L and tristetraprolin, and involvement of AU-rich elements. Nucleic Acids Res. 2012; 40:7739–7752. 10.1093/nar/gks545. 22718976 PMC3439922

